# Avian Reoviruses From Wild Birds Exhibit Pathogenicity to Specific Pathogen Free Chickens by Footpad Route

**DOI:** 10.3389/fvets.2022.844903

**Published:** 2022-02-24

**Authors:** Yu-Ri Choi, Sang-Won Kim, Ke Shang, Jong-Yeol Park, Jun-feng Zhang, Hyung-Kwan Jang, Bai Wei, Se-Yeoun Cha, Min Kang

**Affiliations:** Department of Veterinary Infectious Diseases and Avian Diseases, College of Veterinary Medicine and Center for Poultry Diseases Control, Jeonbuk National University, Iksan, South Korea

**Keywords:** avian reovirus, pathogenicity, non-adaptation, wild bird, carrier, risk, chicken

## Abstract

Avian reoviruses (ARVs) are ubiquitous in domestic poultry with 80% of them being non-pathogenic and they are frequently found in clinically healthy birds. ARVs have also been known to be the etiological agents of viral arthritis (VA), tenosynovitis, myocarditis, runting-stunting syndrome (RSS), and respiratory and enteric disease in chickens. Significant economic losses during the process of poultry husbandry are due, in part, to unmitigated ARV infections throughout the poultry industry. Recently, many isolates shared genetic similarities between those recovered from wild birds and those recovered from poultry. One explanation may be that there is a degree of spillover and spillback of ARVs between the two groups. However, studies on the role of wild birds in the epidemiology and pathogenicity of ARVs are insufficient. Here, we describe the pathogenicity in specific pathogen-free (SPF) chickens of ARV originating from wild birds. The challenge experiment was conducted in six groups including a negative control group, a positive control group (reference strain of S1133), and four groups (A15-157, A18-13, A18-205, A19-106) infected with ARVs from wild birds. The 7-day-old SPF chickens were inoculated with 10^6^TCID_50_ ARV to evaluate the clinical signs, changes in weight gain, gross lesions, histological changes, virus replication, and serum antibody levels. The peak of clinical signs was from 3 to 5 days post infection (dpi). In addition, the death of one chicken was found in the group infected with the A18-13 isolate. Reduced body weight was also found in chickens infected with ARVs from wild birds compared to the negative control group. All the ARVs infection groups showed noticeable swelling of the footpad. In addition, ARVs were detected in the bursa, tendon, and hock joint by reverse transcription-polymerase chain reaction (RT-PCR) in all infected groups at 5 and 15 dpi. Histopathological observations revealed acute inflammatory responses on the synovium covering the joint surfaces (arthritis) and tendon sheaths (tenosynovitis), as well as bursa atrophy and lymphocyte depletion. The analysis of the humoral response was performed by ELISA assay, and chickens infected with ARVs showed seroconverted. In conclusion, this study described the typical severe disease of acute VA and tenosynovitis in SPF chickens infected with ARVs derived from wild birds. This study confirmed the pathogenicity of ARVs infection in SPF chickens for the first time, and these results enrich our understanding of the pathogenicity of ARVs derived from wild birds.

## Introduction

Avian reoviruses (ARVs) are part of the Reoviridae family in the genus *Orthoreovirus*. They are non-enveloped viruses composed of two concentric icosahedral capsids with an external diameter of 80–85 nm (1). The genus *Orthoreovirus* (family Reoviridae) officially contains five virus species (2). *Orthoreoviruses* of birds are classified into the species *Avian orthoreovirus* and into tentative species, such as Tvärminne avian orthoreovirus and Bulbul orthoreovirus (3). The species *Avian orthoreovirus* include all currently known reovirus strains from chicken, turkey, and waterfowl species.

ARVs were first isolated in the United States, in 1957 from synovitis-infected broilers (4). ARVs usually cause low weight, diminished marketability, and high mortality rates, which in turn have economic impacts on the poultry industry. In broilers and turkeys, ARVs infection can cause viral arthritis (VA)/tenosynovitis, which is characterized by swelling of the hock joints and lesions in the gastrocnemius tendons (5–8). Mortality from severe infection can be due to aortic ruptures and the euthanasia of lame chickens (9). ARVs also are the etiological agents of runting-stunting syndrome (RSS) that is characterized clinically by growth retardation, lameness, poor feathering, and shank depigmentation. ARVs are associated with other avian diseases, such as hepatitis, myocarditis, hydropericardium, osteoporosis, malabsorption syndrome (MAS), immunosuppression, respiratory and enteric diseases, and neurological signs (incoordination, tremors, twisted necks) (5, 8, 10–15). Breeder flocks that develop VA during egg production may be characterized by lameness, increased mortality, decreased egg production, and suboptimal hatchability/fertility (1). ARVs are transmitted by the horizontal and vertical routes (16). They affect mostly young birds and are disseminated through the oral-fecal route (17) and vertically from breeders to progeny (18). Transmission to other birds inhabiting a contaminated environment is possible because infected birds excrete significant amounts of the virus in their droppings (8, 17, 19). ARVs survive for at least 10 days on feathers, wood shavings, eggshells, feed, and in drinking water, the virus was detectable for at least 10 weeks (20).

In wild birds, mortalities and responsible for the deterioration in American Woodcock (*Scolopax minor*) were identified to an ARV by Doherty et al. (21). An enteric disease in Bobwhite Quail (*Colinus virginianus*), which resulted in increased mortality in birds. A reovirus was isolated from the feces, and intestinal cryptosporidia were also present (22). Moreover, ARV infection was associated with diarrhea in Pheasant (*Phasianus colchicus*) and neurological signs in a Hooded Crow (*Corvus corone cornix*) (23, 24). These ARVs isolated from wild birds were somewhat genetically distant from those from chicken farms (3, 25). However, recent research showed that ARVs detected in healthy Ostrich (*Struthio camelus*) and a free-living Magpie (*Pica pica*), were found to be genetically related to chicken origin reoviruses (26, 27). Moreover, ARVs from migratory birds were found to be similar to ARVs from chicken farms (28). Little is known about the epizootiology of reoviruses in wild bird populations, and little is known about the wild birds originating ARV infections in chickens. Given the limited information in the literature concerning the pathogenicity of ARV isolates recovered from wild birds, in this study, we evaluated the pathogenicity of ARVs from wild birds in specific-pathogen-free (SPF) 7-day-old chickens.

## Materials and Methods

### Virus Background

The ARV isolates (A15-157, A18-13, A18-205, A19-106) were isolated from fecal samples of wild birds from 2015 to 2019 in South Korea. The A15-157, A18-13, A18-205, and A19-106 isolates were obtained from the Oriental Turtle Dove (*Streptopelia orientalis*), Eurasian Teal (*Anas crecca*), Mallard (*Anas platyrhynchos*), and Bean Goose (*Anser fabalis*), respectively (Table 1). In a previous study, 10 ARVs were isolated from wild bird feces, and all belonged to genotypic cluster I (Supplementary Figure 1) (28). Three isolates (A15-157, A18-205, and A19-106) were selected in each study year (2015, 2018, and 2019), and the A18-13 isolate was selected because it has the same deduced amino acid substitutions with attenuated vaccine S1133 strain and Chinese vaccine break isolates (SD09-1, LN09-1, and GX110116) in σC-encoding gene.

**Table 1:** Isolates of ARV originating from wild birds and a vaccine used in the present study.

**No**.	**Isolate**	**Year**	**Host**	**Genotypic cluster**	**GenBank accession number**
					**S1**
1	A15-157	2015	Oriental Turtle Dove (*Streptopelia orientalis*)	I	MW357868
2	A18-13	2018	Eurasian Teal (*Anas crecca*)	I	MW357869
3	A18-205	2018	Mallard (*Anas platyrhynchos*)	I	MW357871
4	A19-106	2019	Bean Goose (*Anser fabalis*)	I	MW357872
5	S1133	1971	Broiler chickens	I	KF741762

### Virus Isolation

The isolates were inoculated into the chicken embryo liver (CEL) cell culture prepared from 14-day-old specific-pathogen-free (SPF) chicken embryos (Sunrise Farms, Inc. USA). Cell monolayers were infected with 0.2 mL of 10-fold virus fluid and incubated at 37°C for 60 min. Then, a maintenance medium containing 4% fetal bovine serum (FBS) was added. The cultures were incubated at 37°C under 5% CO_2_ and were observed daily under a microscope to check for a cytopathic effect (CPE). Once 70–80% CPE had developed, the cultures were subjected to three cycles of freezing and thawing and then dispensed into a 6-well cell culture plate (SPL Life Sciences, Pocheon, South Korea). The medium for the CEL cell culture was Eagle's minimum essential medium supplemented with 8% FBS and 1% of 100x antibiotic–antimycotic. When the cell monolayers were approximately 80% confluent, the medium was clarified with low centrifugation at 600 × *g* for 20 min (29). After 2 or 3 passages, ARVs were titrated using a 50% tissue culture infective dose (TCID_50_) in 96-well cell culture plates (SPL Life Sciences, Pocheon, Korea). The TCID_50_ was determined in triplicate using the method of Reed and Muench (30). Cells were seeded in 96-well plates and cultured in CEL cell supplemented with 8% FBS until approximately 90% (3.5 x 10^5^/wells) confluent. The ARV isolates generation cells cultures were each diluted 10-fold in a serial dilution in CEL washing buffer (10^−1^-10^−10^). Then supernatant was removed and then incubated with 100 μL suspensions of each dilution of these isolates. The cells in the control group were inoculated with CEL washing buffer (100 μL/well). After incubation at 37°C for 1 h, the CEL supplemented with 4% FBS was used to maintain the normal growth. After, they were incubated in 5% CO_2_ for 3 days at 37°C.

### Detection of Viral Nucleic Acid by RT-PCR and PCR

To screen for potential pathogens, viral DNA or RNA was extracted from the filtered fecal samples. Total viral DNA/RNA was extracted using the Viral Gene-spinTM Viral DNA/RNA Extraction Kit (iNtRON, Seoul, South Korea) according to the manufacturer's instructions. Viral DNA/RNA was used for polymerase chain reaction (PCR) or reverse transcription-PCR (RT-PCR) to detect potential pathogens such as influenza virus, infectious bursa disease virus, paramyxovirus, chicken anemia virus and other common chicken viruses, duck enteritis virus, duck hepatitis virus, duck parvovirus, Tembusu virus, circovirus, and adenovirus (31–33).

In the RT reaction, 8 μL of extracted RNA and 2 μL of dimethyl sulfoxide (DMSO, Tedia, USA) were heated at 100°C for 5 min and placed in an ice bath for 5 min. Then, the following was added to this reaction mixture: 8 μL of GoScriptTM 5x RT reaction buffer (Promega, Madison, WI USA), 10 μL of 2.0 mM of each dNTP (SolGent, Daejeon, South Korea), 4 μL of MgCl_2_ (Promega, Charbonnie‘re, France), 1 μL of 20 units Recombinant RNase Ribonuclease Inhibitor (Promega, Madison, WI, USA), 1 μL of GoScriptTM reverse transcriptase, 1 μL of 50 pmoL random primers, and 4 μL of diethylpyrocarbonate-treated water (DEPC water; Biosesang, Seoul, South Korea); a final volume of 39 μL was obtained.

The DNA or cDNA (5 μL) was specifically amplified with PCR. The reaction (total volume of 50 μL) contained 5 μL of 10x e-Taq buffer (SolGent, Daejeon, South Korea), 5 μL of 2.0 mM dNTP (SolGent, Daejeon, South Korea), each of the upstream and downstream primers for target gene (5 pmol), 0.25 μL of the Solg^TM^ e-Taq and 31.75 μL of the ddH_2_O.

For the detection of ARVs from fecal samples as well as the CEL cell culture fluid stocks, the partial S1 gene was amplified. Thermal cycling protocols were as follows: initial denaturation at 94°C for 5 min, 35 cycles (denaturation at 94°C for 1 min, annealing at 50°C for 1 min, and extension at 72°C for 1 min), and one final extension at 72°C for 10 min (34). For the sequence analysis, viral cDNA was obtained from RNA samples using the above-mentioned method.

### Animal Experiments

Forty-eight 1-day-old SPF white leghorn chicks were purchased (Namduk Company, Gyeonggi, South Korea). The chicks were kept in separate negative-pressure isolators animal care units for 7 days to adapt and were provided with food and water *ad libitum*. The chicks were wing-banded individually and reared under uniform management care in an isolator. Before starting the experiments, the chick's ELISA results showed that there were not ND, AI, ARVs, and other common chicken viruses in the serum. Seven-day-old chickens were divided into six groups of eight chickens for pathogenicity analysis and survival analysis. Four of the groups were infected by the right footpad route (35) by inoculation with 10^6^TCID_50_/0.1 mL of the A15-157, A18-13, A18-205, and A19-106 wild bird isolates, respectively. Commercial vaccine strain S1133 was used as a positive control, and sterile phosphate-buffered saline [PBS, pH7.4; supplemented with 100x antibiotic-antimycotic (Gibco, New York, USA)] was used as a negative control. The positive control group was inoculated with 10^6^TCID_50_/0.1 mL of S1133 strain. The negative control group was inoculated with PBS. Chickens were weighed at 0, 3, 5, 7, 9, and 14 days post-inoculation (dpi).

The behavior of the infected chickens and their gross external and internal signs were recorded during the experimental infection period. General body condition was evaluated by clinical signs. Clinical signs were observed at 1, 3, 5, 7, 9, and 14 dpi. Swelling, redness, depression, and lameness were assigned scores of 0, 1, 2, or 3 for each chicken. The swelling of the footpad was visually scored as follows: 0, normal; 1, plantar metatarsal or digital pads with swellings; 2, pads and interdigital web swelling; 3, metatarsal area swelling. Gastrocnemius tendon and intertarsal joints were scored as follows: 0, normal; 1, mildly swelling; 2, moderately swelling; 3, severely swelling. Redness was scored as follows: 0, normal; 1, only foot; 2, foot and leg; 3, all of them. Depression scores were determined for the observed signs: 0, active; 1, low responsiveness to sound stimulation; 2, stand still but respond to sound stimulation; and 3, no response to sound stimulation. Lameness scores were determined for the observed signs: 0, normal; 1, slight limp (or inconsistent); 2, major lameness, unwilling to walk; and 3, standing on one leg or sitting, refuses to walk. Three chickens from each group were drawn randomly for necropsy at 5 and 15 dpi. The chickens were euthanized, necropsied, and examined for the presence of gross lesions, and the pancreas, bursa, and arthrosis-affected tissues were collected. Sections of the affected pancreas, bursa, gastrocnemius tendon, and intertarsal joint tissues were fixed in 10% neutral buffered formalin. The fixed tissues were embedded in paraffin, sectioned at 5 μm thickness, stained with hematoxylin and eosin, and examined under a light microscope. Other tissues were stored at −70°C until use for RNA extraction. All experimental and animal management procedures were undertaken following the requirements of the Animal Care and Ethics Committee of Jeonbuk National University. The animal facility at Jeonbuk National University is fully accredited by the National Association of Laboratory Animal Care (approval number: JBNU 2021-0139).

### Identification of Virus in Organs

The RT-PCR was carried out in tissue samples obtained from chickens to detect ARVs. Samples were obtained from the tendon, hock joint and bursa of Fabricius. The primers MK87 (5′-GGTGCGACTGCTGTATTTGGTAAC-3′) and MK88 (5′-AATGGAACGATAGCGTGTGGG-3′) were used to amplify the partial S1 gene of ARVs (34).

### Enzyme-Linked Immunosorbent Assay

Serum samples were collected from chickens infected with ARVs (A15-157, A18-13, A18-205, and A19-106 isolates and S1133 strain) on 14 and 21 dpi to measure the antibody response. Complement in serum samples had to be inactivated at 56°C for 30 min. ARV antibodies in the sera were determined by a commercial indirect ELISA test kit (BioChek, Holland) (6).

### Statistical Analysis

The experimental test data are expressed as means ± standard deviations (SDs). Statistical calculations were made with the SPSS programming tool (IBM SPSS. 20®) (SPSS Inc., Chicago, IL, USA) using one-way ANOVA followed by Tukey's *post-hoc* test. Statistical significance was defined as *P* < 0.05.

## Results

### Clinical Signs and Body Weight Changes

**Figure 1:**
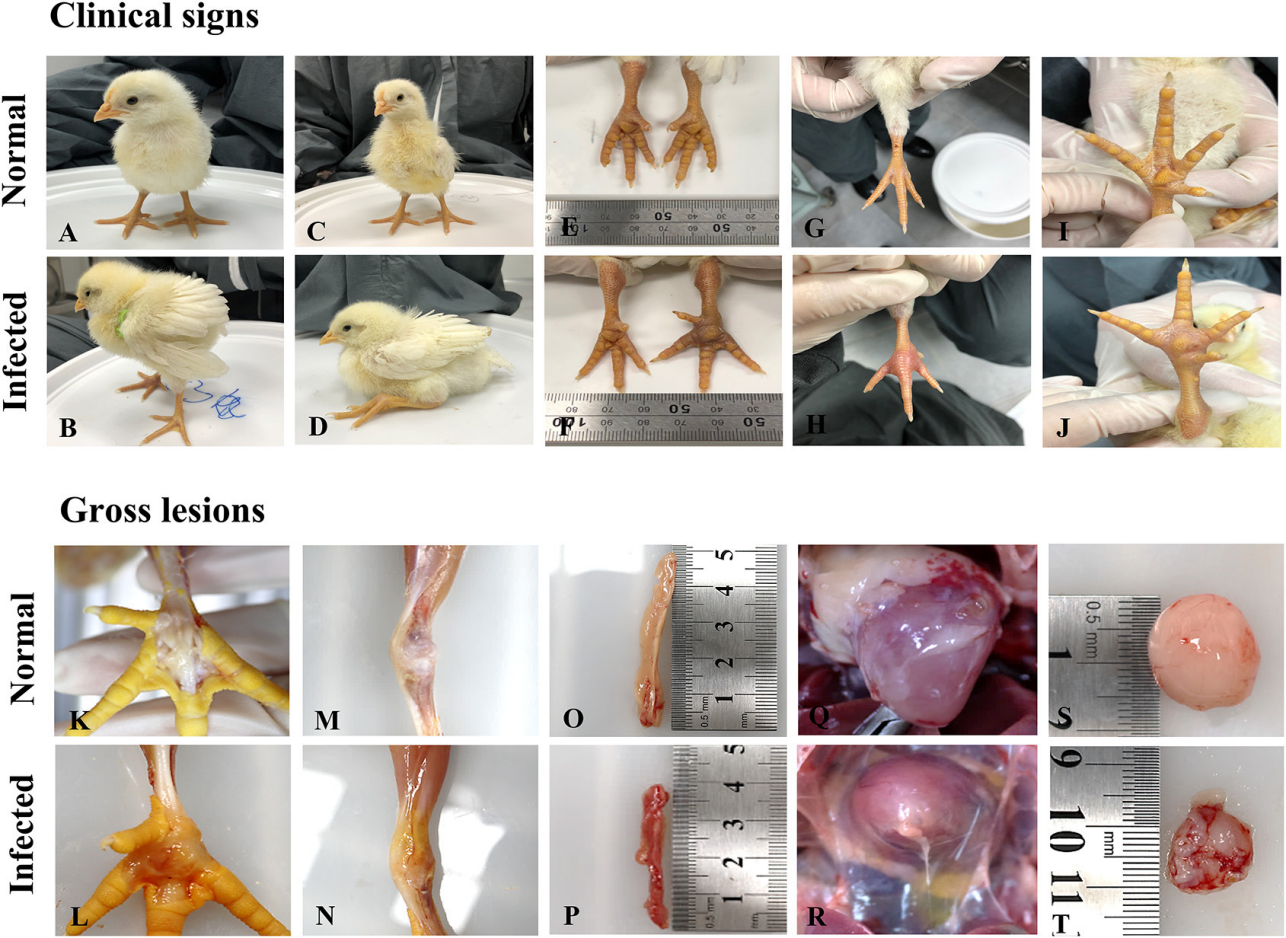
Clinical signs and pathological changes in SPF chicken after ARV isolates originating from wild birds **(A)** The control group was normal; **(B)** Chickens displaying depression, drowsiness, and fluffy feathers at 3 dpi; **(C)** The control group was normal; **(D)** Chickens displaying lameness at 3 dpi; **(E)** The leg of chickens in the control group was normal; **(F)** Tenosynovitis associated with the entire right leg 4 dpi; **(G)** The foot of chickens in the control group was normal; **(H)** Severe redness in the foot at 4 dpi; **(I)** The footpad of chickens in control group was normal; **(J)** Severe swelling in the footpad at 4 dpi; **(K)** The footpad of chickens in the control group was normal; **(L)** Swelling, edema, and hemorrhages in the footpad at 5 dpi; **(M)** The articular cavity of chickens in the control group was normal; **(N)** Swelling and edema in the articular cavity; **(O)** The pancreas of chickens in the control group was normal; **(P)** Pancreas with atrophy and hemorrhage at 5 dpi; **(Q)** The heart of chickens in the control group; **(R)** Heart with edema and hydropericardium at 15 dpi; **(S)** The bursa of chickens in the control group was normal; **(T)** Moderate atrophy and hemorrhages in the Bursa of Fabricius.

**Figure 2:**
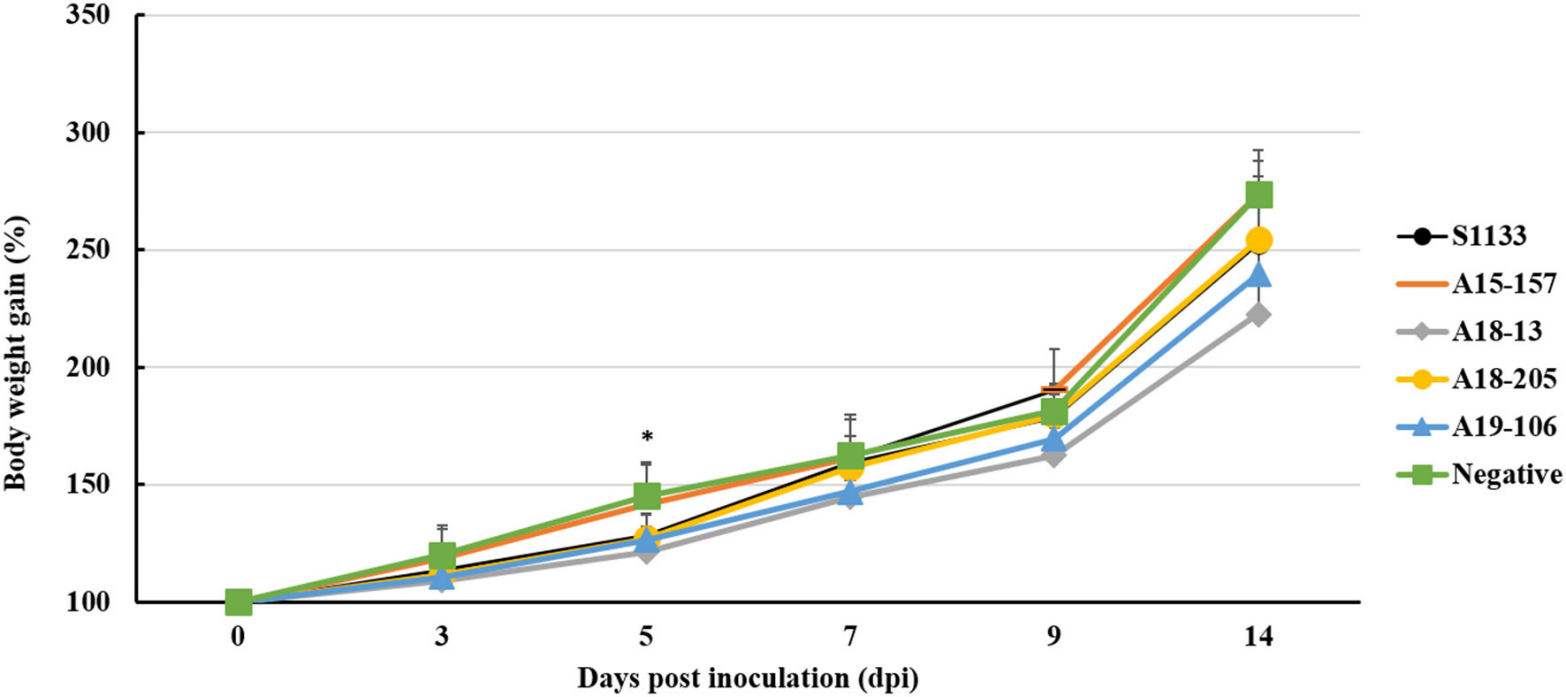
Body weight changes of chickens infected with ARV by footpad route. The ARV-infected groups were infected with 10^6^ TCID_50_/0.1 mL of ARV. The negative control group was inoculated with 0.1 ml PBS. Bars show mean ± SD. The mean value was statistically significant, determined using one-way ANOVA followed by Tukey's *post-hoc (P* < 0.05). Asterisk (*) indicates *p* < 0.05 and represents a significant difference between the negative control (PBS) group and virus-infected groups.

The S1133 strain infected chickens began to exhibit depression, anorexia, half-closed eyes, and dozing off at 1 dpi (Table 2). The peak of clinical signs included skin redness in the foot and tendon, swelling of the footpad, tendon, and hock joint from 3 to 5 dpi. In addition, lameness, and splayed leg due to tenosynovitis spanning the femoro-tibiotarsal and intertarsal joints and plantar metatarsal region were observed at 1 dpi which persisted till 14 dpi. Clinical signs were observed in chickens infected with ARVs at 1 dpi, which gradually increased to a peak at 3–5 dpi (Supplementary Table 1). Thereafter, the clinical signs decreased but persisted up to 14 dpi. The A19-106 isolate infected chickens showed similar clinical signs and peak as the S1133 strain infected chickens. Compared to the S1133 strain, the A15-157 isolate infected chickens exhibit mild clinical signs of skin redness in the foot and tendon and swelling of the hock joint; the A18-205 isolate infected chickens exhibited mild clinical signs in skin redness in the foot and tendon, but chickens exhibited severe in swelling of the hock joint; the A18-13 isolate infected chickens exhibit severe clinical signs of skin redness in the foot and tendon, swelling of the footpad, tendon, and hock joint, and lameness (Figure 1). One chicken infected with the A18-13 isolate died at 4 dpi. No chickens infected with the A15-157, A18-205, A19-106 isolates, or S1133 strain died. No clinical manifestations were observed in the negative control groups. Each chicken was randomly selected from each group and weighed at 0, 3, 5, 9, and 14 dpi. The groups infected with ARVs (A15-157, A18-205, A19-106 isolates, and S1133 strain) showed a decrease in body weight compared with the negative control group. The chickens infected with the A18-13 isolate were statistically significantly lighter in mass when compared to the negative control group at 5 dpi (*P* < 0.05) (Figure 2). Conversely, mean body weights were statistically indistinguishable among the groups at 7, 9, and 14 dpi.

**Table 2:** The clinical manifestation of chicken infected with ARV originating from wild birds.

	**Clinical manifestation**					
**Isolate**	**1 dpi**	**3 dpi**	**5 dpi**	**7 dpi**	**9 dpi**	**14 dpi**
PBS	Normal	Normal	Normal	Normal	Normal	Normal
S1133	Skin redness (7/8), Footpad swelling (8/8), Tendon swelling (5/8), Hock joint swelling (3/8), Lameness (8/8)	Skin redness (8/8), Footpad swelling (8/8), Tendon swelling (8/8), Hock joint swelling (6/8), Lameness (8/8)	Skin redness (8/8), Footpad swelling (8/8), Tendon swelling (8/8), Hock joint swelling (7/8), Lameness (8/8), depression (1/8)	Skin redness (5/5), Footpad swelling (5/5), Tendon swelling (5/5), Hock joint swelling (3/5), Lameness (5/5), depression (1/5)	Skin redness (4/5), Footpad swelling (5/5), Tendon swelling (4/5), Hock joint swelling (2/5), Lameness (5/5), depression (1/5)	Skin redness (4/5), Footpad swelling (5/5), Tendon swelling (4/5), Lameness (3/5)
A15-157	Skin redness (8/8), Footpad swelling (8/8), Tendon swelling (6/8), Hock joint swelling (2/8), Lameness (8/8)	Skin redness (8/8), Footpad swelling (8/8), Tendon swelling (8/8), Hock joint swelling (6/8), Lameness (8/8), depression (1/8)	Skin redness (2/8), Footpad swelling (8/8), Tendon swelling (8/8), Hock joint swelling (6/8), Lameness (8/8)	Footpad swelling (5/5), Tendon swelling (4/5), Hock joint swelling (1/5), Lameness (5/5)	Footpad swelling (5/5), Tendon swelling (3/5), Hock joint swelling (1/5), Lameness (5/5)	Footpad swelling (5/5), Lameness (1/5)
A18-13	Skin redness (8/8), Footpad swelling (8/8), Tendon swelling (8/8), Hock joint swelling (4/8), Lameness (8/8)	Skin redness (8/8), Footpad swelling (8/8), Tendon swelling (8/8), Hock joint swelling (8/8), Lameness (8/8)	Skin redness (7/7), Footpad swelling (7/7), Tendon swelling (7/7), Hock joint swelling (7/7), Lameness (7/7), depression (3/7)	Skin redness (4/5), Footpad swelling (5/5), Tendon swelling (5/5), Hock joint swelling (5/5), Lameness (5/5), depression (2/5)	Skin redness (4/5), Footpad swelling (5/5), Tendon swelling (5/5), Hock joint swelling (5/5), Lameness (5/5), depression (2/5)	Skin redness (4/5), Footpad swelling (5/5), Tendon swelling (5/5), Hock joint swelling (5/5), Lameness (2/5), depression (2/5)
A18-205	Skin redness (6/8), Footpad swelling (8/8), Tendon swelling (6/8), Hock joint swelling (0/8), Lameness (8/8)	Skin redness (8/8), Footpad swelling (8/8), Tendon swelling (8/8), Hock joint swelling (3/8), Lameness (8/8)	Skin redness (6/8), Footpad swelling (8/8), Tendon swelling (8/8), Hock joint swelling (7/8), Lameness (8/8)	Skin redness (2/5), Footpad swelling (5/5), Tendon swelling (5/5), Hock joint swelling (5/5), Lameness (5/5)	Skin redness (2/5), Footpad swelling (5/5), Tendon swelling (5/5), Hock joint swelling (2/5), Lameness (5/5)	Skin redness (2/5), Footpad swelling (5/5), Tendon swelling (5/5), Hock joint swelling (2/5)
A19-106	Skin redness (6/8), Footpad swelling (8/8), Tendon swelling (2/8), Hock joint swelling (0/8), Lameness (8/8)	Skin redness (8/8), Footpad swelling (8/8), Tendon swelling (7/8), Hock joint swelling (7/8), Lameness (8/8), depression (1/8)	Skin redness (8/8), Footpad swelling (8/8), Tendon swelling (7/8), Hock joint swelling (8/8), Lameness (8/8), depression (2/8)	Skin redness (4/5), Footpad swelling (5/5), Tendon swelling (5/5), Hock joint swelling (4/5), Lameness (5/5)	Skin redness (4/5), Footpad swelling (5/5), Tendon swelling (5/5), Hock joint swelling (2/5), Lameness (2/5)	Skin redness (4/5), Footpad swelling (5/5), Tendon swelling (5/5), Hock joint swelling (2/5)

### Gross Lesions

At 5 and 15 dpi, three chickens from each group were randomly euthanized and drawn for necropsy evaluation. The gross lesions in the S1133 infected chickens at 5 dpi showed moderate swelling, edema, and hemorrhages in the tendons, and inflammation of the footpad extending up to the hock joint including the synovial membranes and the surrounding tissue. In addition, the liver showed severe swelling, the bursa of Fabricius showed moderate inflammation, the heart showed mild peripheral edema, and the pancreas showed atrophy and severe multiple punctate hemorrhage foci. Lesions in tendons, footpad, hock joint, and liver were less pronounced at 15 dpi but showed severe lesions in the pancreas and bursa of Fabricius compared to at 5 dpi. Compared to the S1133 strain, the gross lesions in the A18-205 isolate infected chickens at 5 dpi showed mild lesions in the liver and pancreas. All gross lesions at 15 dpi were less pronounced than at 5 dpi. In the A15-157 isolate, there were no lesions in the pancreas, and mild lesions in tendons, footpad, and hock joint compared to the S1133 strain at 5 dpi, and all gross lesions at 15 dpi were less pronounced than 5 dpi. Compared to the S1133 strain, the gross lesions in the A19-106 isolate infected chickens at 5 dpi showed severe lesions in the liver, tendons, and footpad, and no lesions in the heart, but severe hydropericardium at 15 dpi; the gross lesions in the A18-13 isolate infected chickens at 5 dpi showed severe lesions in the liver, kidney, and pancreas (Figure 1). Hepatomegaly and friability were seen in the liver, and there were many yellowish-white focal necroses of variable size on the surface or in the parenchyma of the liver. The kidney was swollen and showed hemorrhages, and the pancreas showed atrophy and severe multiple punctate hemorrhage foci. No gross lesions were observed in the negative control group.

### Histological Lesions

**Figure 3:**
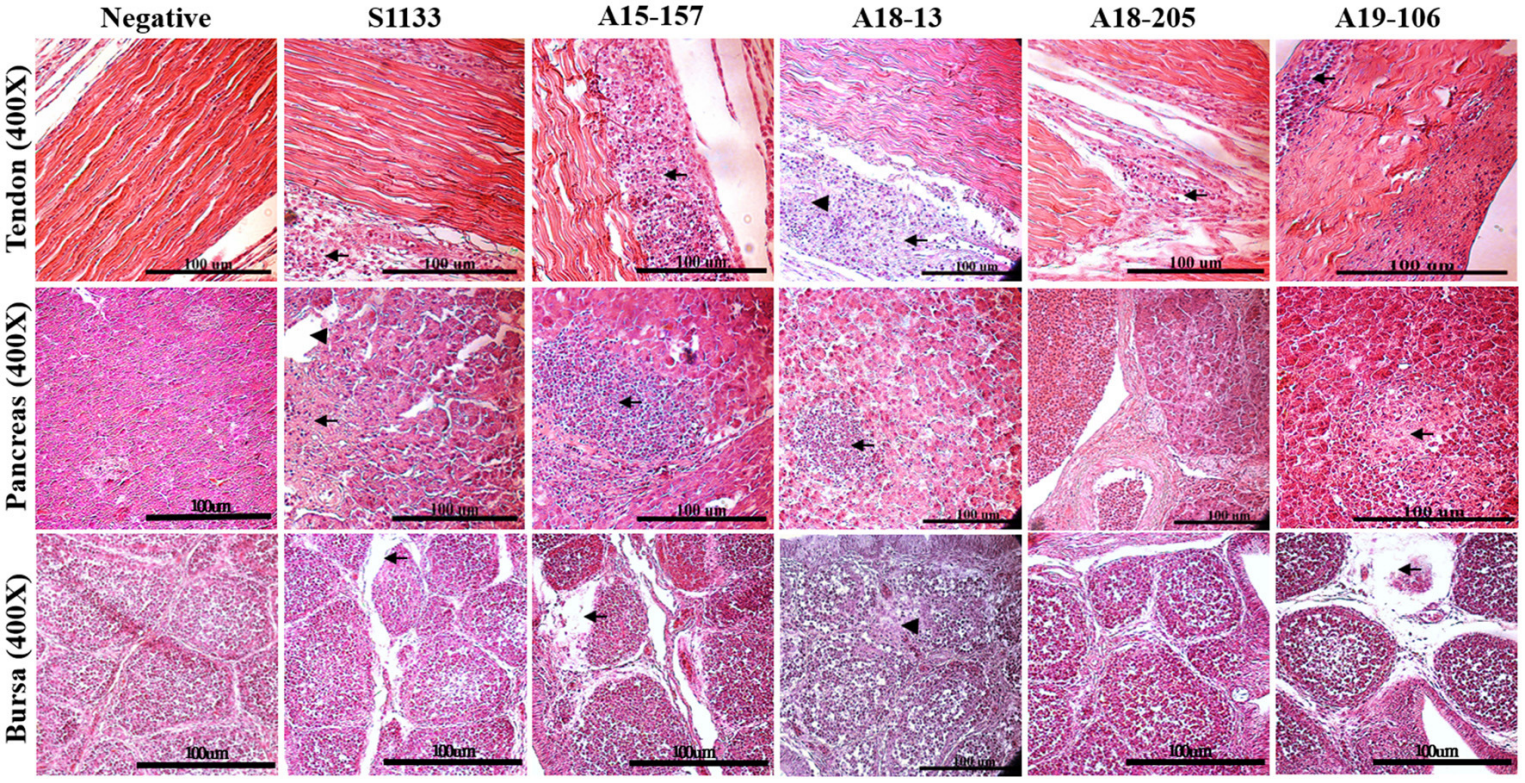
Histopathological changes in chickens infected with ARVs. Pathological changes including subsynovial lymphocytic infiltration (arrow) and fibrosis (arrowheads) were found in the tendon section; extensive inflammatory cells (arrows), dilated acini (arrowheads), and hemorrhage were found in the pancreas section. Lymphocyte depletion in the bursa section (arrow) and heterophilic granulocyte infiltration in the cortex (arrowheads) were found in those infected with ARV but not in the negative control group (PBS).

The histopathological changes of the chickens in the ARV-infected groups were similar, appearing in the gastrocnemius tendons, pancreas, and bursa of Fabricius, as in the S1133 positive control (Figure 3). The lesions were observed in the gastrocnemius tendons. There was not only tendon fibrosis but also an acute inflammatory response involving the tendon sheaths (tenosynovitis), with infiltration of inflammatory cells. Other microscopic lesions manifested as fibroplasia and inflammatory cell infiltration in the synovial cavity, massive loss of lymphocytes from the bursa of Fabricius, and pancreatic cell disintegration with infiltration of inflammatory cells in the pancreas. No histopathological lesions were observed in the negative control group.

### Detection of ARV Gene in Different Organs

Pooled tissues from different organs of chicken carcasses were tested by RT-PCR at 5 and 15 dpi to investigate the replication ARVs in the tendon, hock joint, and bursa of Fabricius. All samples were positive at 5 and 15 dpi in all ARVs infected groups.

### Detection of Serum Antibody Titers

As seen in Supplementary Table 2, sera from chickens infected with ARVs (A15-157, A18-13, A18-205, A19-106 isolates, and the S1133 strain) were collected at 14 and 21 dpi, and tested with an ELISA. From our results, chickens infected with ARVs were seroconverted at 14 dpi. From 14 dpi, the serum antibody titers were continuously ascending, and the level at 21 dpi reached a much higher level. No antibodies against ARV were observed in the negative control group.

## Discussion

Significant economic losses in poultry husbandry due to ARV infections emphasize the importance of continuously studying the prevalence, genetic characterization, and evolution of the pathogenicity of the newly emerging ARV isolates in the poultry industry. ARVs have also been identified in wild birds, such as Hooded Crows (*Corvus corone cornix*), Magpies (*Pica pica*), Partridges (*Perdix perdix*), Black-Capped Chickadees (*Poecile atricapillus*), Brown-Eared Bulbuls (*Hypsipetes amaurotis*), Psittacine bird species (*Psittacus erithacus*), and Mallards (*Anas platyrhynchos*) (2, 25). The genetic similarities of ARVs between isolates from wild birds and poultry suggest the circulation of ARVs between wild birds and poultry. These viruses can infect both wild birds as well as poultry (2, 26). Moreover, in particular, wild migratory birds that could migrate within and across continents; and wild birds are the reservoirs for the virus gene pool of avian influenza virus and avian paramyxovirus-1 (36). However, studies on the role of wild birds in epidemiology, and the pathogenic characterization of ARVs from wild birds are insufficient. ARVs are widespread and could affect various commercial and wild avian species. ARVs have been isolated from poultry, such as chickens, ducks, turkeys, ostriches, and wild birds (2, 37). In previous studies, wild bird-origin ARVs were found to belong to the Tvärminne avian orthoreovirus (TVAV)-like cluster (3, 24, 25, 38, 39). These ARVs isolated from wild birds were somewhat genetically distant from those at chicken farms (3, 25). Recently, research showed that the σA-encoding gene of ARVs isolated from a healthy ostrich at a domestic farm in Japan had great similarity to the chicken-origin ARVs, and the σC-encoding gene isolated from magpies was found to be genetically similar to the chicken-origin ARVs (26). Based on the σC protein sequence, the phylogenetic analysis revealed that our isolates from wild birds belonged to GC I, which is the predominant cluster in chicken-origin ARVs (28). This includes commercial vaccine isolates and induces the production of the disease in chickens by experimental infection (8, 40). Genetic similarities of ARVs from wild birds and poultry support the hypothesis of circulation of ARVs between wild birds and poultry. However, experimental infections with the ARV isolates from wild birds were not conducted.

ARV pathogenicity is very variable from high to low virulence (41). ARV isolates in domestic poultry with 80% of them being non-pathogenic and are frequently found in clinical healthy birds (17, 42, 43). ARV infection is associated with transitional ARV diseases, including arthritis/tenosynovitis, MAS, RSS, respiratory diseases, hepatitis, myocarditis, and immunosuppression (44, 45). Here, we described the pathogenicity in chickens of ARV originating from wild birds. All chickens experimentally infected with isolates derived from wild birds showed depression, anorexia, lameness, joint disease, and similar pathogenic damage in the hock joint and tendon to those with the S1133 strain infection (36, 41, 46). ARV isolates caused a disease typical of acute VA and tenosynovitis (Figure 1) that recovered after 14 dpi. The results of chicken weight loss showed that the growth of the chickens infected with the ARV isolates was inhibited compared to the negative control group, and the growth of chickens infected with A18-13 was significantly inhibited (Figure 2). This indicates that chickens infected with the wild bird ARV isolates suffered growth retardation or malabsorption, which is consistent with previous reports on ARV-infected chickens with RSS and MAS (47). The pathological changes of the chickens in the four experimental groups were similar to those of the S1133 positive control, and the main difference was the severity of pathological manifestations. The gross pathological lesions included marked swelling, edema, hemorrhages, and serous exudate between tendons. The tenosynovitis observed in the chickens inoculated with the wild bird ARV isolates was similar to that described by others (6). In experimental studies, chickens inoculated with reovirus *via* the footpad route usually have edema and acute inflammatory cell infiltration in the peritendon sheath within 2–7 days of infection (6, 7). In our study, we observed early onset of acute tenosynovitis on day 1 following footpad inoculation with all ARV isolates of wild birds (Table 2) suggesting that these isolates may have a strong arthrotropic potential in chickens. Systemic infection by all ARV isolates of wild birds was evident with the presence of pericarditis, hepatic necrosis, severe multiple punctate hemorrhage foci on the pancreas, and bursal atrophy in chickens. The characteristic tenosynovitis together with these clinical signs has been suggested as diagnostic criteria for VA caused by ARV. It was found that ARV isolated from wild birds produced gross pathological lesions in chickens similar to ARVs from chickens previously (27, 36).

The histological lesions seen in the present study were also similar to those observed in other investigations of ARV infection in chickens, demonstrating mixed inflammatory infiltrate in the tendon sheaths and pancreas, while typical lymphocyte depletion was obvious in the bursa of Fabricius (43, 48). Histopathological lesions manifested massive loss of lymphocytes in the bursa of Fabricius and disintegration of pancreatic cells (Figure 3) were found in the infected chickens, which could be the main reason for the reduced immunity of chickens infected with ARV and the secondary infection with other pathogens (6). Compared with the S1133 strain, in terms of incidence and severity, our isolates were equivalently virulent. Viral RNA among different tissues in each group was determined, showing that the virus was detected in the bursa of Fabricius, hock joint, and tendon of SPF chickens, indicating that our isolates had specific tissue tropism, including immune organs. Our ARV isolates from wild birds had a strong erosive ability on the bursa, resulting in dysfunction of the immune system, which may have been the main factor causing the secondary infection of chickens after the initial infection with ARV (48–50). Bursa of Fabricius is a very important immune organs in poultry, pathological damage can lead to the immunosuppression of the body (6). Previous studies have confirmed that the bursa may be the target organ for the initial replication of ARV and that the virus does not replicate efficiently in other tissues, which may be related to its replication mechanism (7, 51–53). In this study, these findings indicated that the ARV isolates from wild birds could infect chickens *via* the footpad and typical severe disease of acute VA and tenosynovitis in SPF chickens.

## Conclusion

This study demonstrated clinical signs, gross and histological features associated with acute VA and tenosynovitis in SPF chickens infected with ARVs from wild birds. To the authors' knowledge, this is the first report of ARV infection associated with significant disease in SPF chickens by ARVs from wild birds.

## Data Availability Statement

The datasets presented in this study can be found in online repositories. The names of the repository/repositories and accession number(s) can be found in the article/Supplementary Material.

## Ethics Statement

The animal study was reviewed and approved by Animal Care and Ethics Committee of Jeonbuk National University (approval number: JBNU 2021-0139).

## Author Contributions

S-YC and MK contributed to the conception and design of experiments. H-KJ, S-WK, Y-RC, KS, BW, J-YP, and J-fZ contributed to the acquisition, analysis, and interpretation of data. S-WK, Y-RC, S-YC, and MK drafted and/or revised the article. All authors have read and agreed to the published version of the manuscript. All authors contributed to the article and approved the submitted version.

## Funding

This work was supported by the Korea Institute of Planning and Evaluation for Technology in Food, Agriculture and Forestry (IPET) through Agriculture, Food and Rural Affairs Convergence Technologies Program for Educating Creative Global Leader (716002-7, 320005-4) funded by the Ministry of Agriculture, Food and Rural Affairs (MAFRA). The funders had no role in study design, data collection, analysis, decision to publish, or preparation of the manuscript.

## Conflict of Interest

The authors declare that the research was conducted in the absence of any commercial or financial relationships that could be construed as a potential conflict of interest.

## Publisher's Note

All claims expressed in this article are solely those of the authors and do not necessarily represent those of their affiliated organizations, or those of the publisher, the editors and the reviewers. Any product that may be evaluated in this article, or claim that may be made by its manufacturer, is not guaranteed or endorsed by the publisher.

## Supplementary Material

Supplementary Figure 1 Phylogenetic tree of ARV isolates based on Sigma C amino acid sequences (326 aa). Maximum likelihood phylogenetic analyses were conducted using MEGA-X software with the Jones Taylor Thornton (JTT) model and 1,000 bootstrap replicates. The tree shows the genetic relationships between the Sigma C protein sequences (326 amino acids) of our four isolates and the 40 reference isolates that were isolated from around the world. The virus isolates are clustered into six GCs. The black circles (•) indicate our isolates, and the white circles (⚬) indicate the vaccine isolates. Additionally, the black diamonds (♦) indicate field isolates in Korea, and the black triangles (▴) indicate previously isolated wild bird isolates. Each sequence on the tree is identified by the isolate name, host, country of origin, year of isolation, and GenBank accession number.

Supplementary Table 1 Scoring for the clinical signs of ARVs infected chickens at 5 dpi.

Supplementary Table 2 Serum antibody titers against ARVs (A15-157, A18-13, A18-205, and A19-106 isolates and S1133 strain).
