# Supplementary material for: Avian Reoviruses From Wild Birds Exhibit Pathogenicity to Specific Pathogen Free Chickens by Footpad Route
Source: Front Vet Sci. 2022 Feb 24;9:844903. doi: 10.3389/fvets.2022.844903 (PMC8907544; doi:10.3389/fvets.2022.844903)
Supplement: Supplementary Figure 1 — Phylogenetic tree of ARV isolates based on Sigma C amino acid sequences (326 aa). Maximum likelihood phylogenetic analyses were conducted using MEGA-X software with the Jones Taylor Thornton (JTT) model and 1,000 bootstrap replicates. The tree shows the genetic relationships between the Sigma C protein sequences (326 amino acids) of our four isolates and the 40 reference isolates that were isolated from around the world. The virus isolates are clustered into six GCs. The black circles (•) indicate our isolates, and the white circles (⚬) indicate the vaccine isolates. Additionally, the black diamonds (♦) indicate field isolates in Korea, and the black triangles (▴) indicate previously isolated wild bird isolates. Each sequence on the tree is identified by the isolate name, host, country of origin, year of isolation, and GenBank accession number. [file Data_Sheet_1.docx]

Supplementary Material

**Supplementary Figure 1**

Phylogenetic tree of ARV isolates based on Sigma C amino acid sequences (326 aa). **
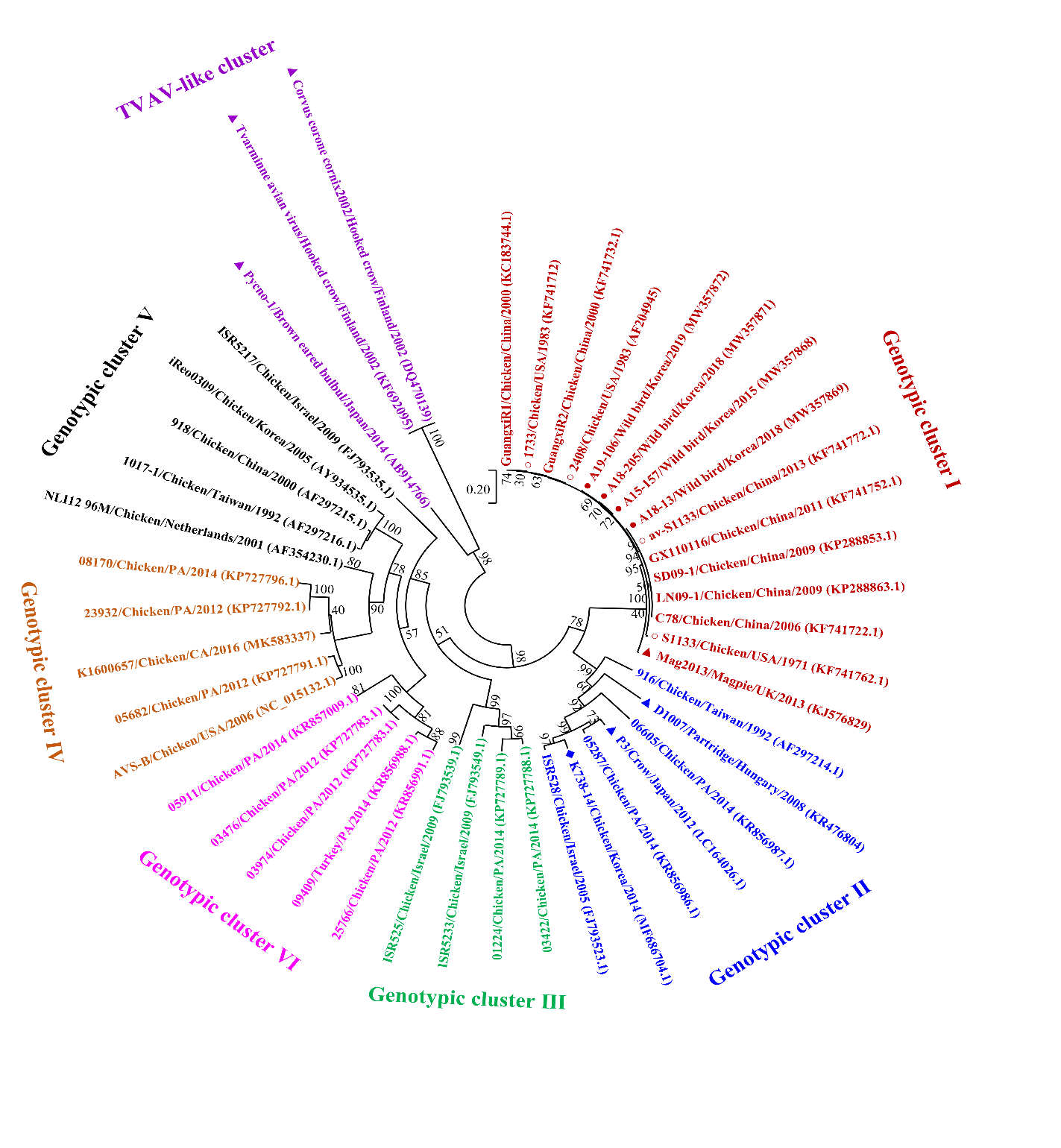
**

**Supplementary Table 1 |** Scoring for the clinical signs of ARVs infected chickens at 5 dpi. ^a^

| Group (*n* = 6) | Score | Clinical sign scoring | | | | | |
| --- | --- | --- | --- | --- | --- | --- | --- |
|  |  | Footpad | Tendon | Joint | Redness | Depression | Lameness |
| PBS | 1 | 0 | 0 | 0 | 0 | 0 | 0 |
|  | 2 | 0 | 0 | 0 | 0 | 0 | 0 |
|  | 3 | 0 | 0 | 0 | 0 | 0 | 0 |
|  | Total | 0 | 0 | 0 | 0 | 0 | 0 |
| S1133 | 1 | 0 | 0 | 4 | 0 | 0 | 8 |
|  | 2 | 0 | 5 | 2 | 4 | 0 | 0 |
|  | 3 | 8 | 3 | 1 | 4 | 1 | 0 |
|  | Total | 24 | 19 | 11 | 20 | 3 | 8 |
| A15-157 | 1 | 0 | 2 | 2 | 1 | 0 | 8 |
|  | 2 | 4 | 4 | 3 | 1 | 0 | 0 |
|  | 3 | 4 | 2 | 1 | 0 | 0 | 0 |
|  | Total | 20 | 16 | 11 | 3 | 0 | 8 |
| A18-13 | 1 | 3 | 2 | 4 | 2 | 0 | 7 |
|  | 2 | 2 | 5 | 3 | 3 | 3 | 0 |
|  | 3 | 3 | 1 | 1 | 3 | 1 | 1 |
|  | Total | 16 | 15 | 13 | 17 | 9 | 10 |
| A18-205 | 1 | 0 | 1 | 7 | 1 | 0 | 8 |
|  | 2 | 0 | 6 | 1 | 5 | 0 | 0 |
|  | 3 | 8 | 1 | 0 | 1 | 0 | 0 |
|  | Total | 24 | 16 | 9 | 14 | 0 | 8 |
| A19-106 | 1 | 0 | 3 | 5 | 0 | 1 | 8 |
|  | 2 | 1 | 4 | 3 | 5 | 1 | 0 |
|  | 3 | 7 | 1 | 0 | 2 | 0 | 0 |
|  | Total | 23 | 14 | 11 | 16 | 3 | 8 |

^a^Clinical signs of ARVs (A15-157, A18-13, A18-205, A19-106 isolates, and S1133 strain) infected chickens were scored into 3 categories, chickens with slight symptoms were scored as 1; those with a moderate symptom was scored as 2, and those with severe symptoms were scored as 3. No abnormal clinical signs were observed in negative control groups at 5dpi. In comparison, chickens in the ARVs infection group showed mild (score 0.4) to moderate clinical signs (score 3.0) at 5 dpi. All chickens in A19-106 and A15-157 showed mild depression signs, scores ranging from 0 to 2 with the highest score in S1133 and A18-13 (score 3). On the other hand, at 5 dpi all infected groups showed moderate to severe footpad swelling with a total score range from 16 to 24.

## Supplementary Table 2

Serum antibody titers against ARVs (A15-157, A18-13, A18-205, A19-106 isolates, and S1133 strain).

| Bleeding | Isolates | | | | | |
| --- | --- | --- | --- | --- | --- | --- |
|  | S1133 | A15-157 | A18-13 | A18-205 | A19-106 |  |
| 14 dpi | 3957±532 ^a^ | 2570±762 | 4523±746 | 3030±309 | 3309±624 |  |
| 21 dpi | 7916±80 | 6965±1161 | 7540±2134 | 6600±500 | 7390±1893 |  |

^a^Data are expressed as mean±SDs.
